# Impact of the COVID-19 pandemic on growth, nutrition and immune function in infants and toddlers

**DOI:** 10.1017/S1368980026102808

**Published:** 2026-06-04

**Authors:** Yingli Lin, Dongshui Kang, Qi Chen, Shuang Chen

**Affiliations:** 1 Department of Early Childhood Education, Shaoxing Vocational & Technical College, China; 2 Department of Pediatrics, Sanming First Hospital Affiliated to Fujian Medical University, China; 3 Department of Pediatrics, Tiantai People’s Hospital of Zhejiang Province, China; 4 Department of Pediatrics, Taizhou Hospital of Zhejiang Province Affiliated to Wenzhou Medical University, China

**Keywords:** COVID-19, Child growth, Nutritional intake, Immune function, NHANES, Paediatric health

## Abstract

**Objective:**

To evaluate the impact of the COVID-19 pandemic on physical growth, nutritional intake and immune function in children aged 12–24 months.

**Design:**

A cross-sectional analysis was conducted utilising data from the National Health and Nutrition Examination Survey (NHANES). Pre-pandemic (2017–2018) and pandemic (2021–2023) cohorts were compared.

**Setting:**

Nationally representative data from the United States.

**Participants:**

Children aged 12–24 months (pre-pandemic: *n* 3 904 863; pandemic: *n* 3 496 904).

**Results:**

No significant differences were observed in physical growth (weight, length and BMI) or macronutrient intake between cohorts. Pandemic-born children had lower vitamin A (437·0 *v*. 540·0 mcg/d, *P* = 0·006) and B_12_ (2·7 *v*. 3·4 mcg/d, *P* = 0·009) intake but higher vitamin C intake (87·0 *v*. 62·8 mg/d, *P* < 0·001). Inflammatory markers (neutrophil-to-lymphocyte ratio, platelet-to-lymphocyte ratio and systemic immune-inflammation index) showed minor age- and sex-specific variations but remained within normal ranges. Environmental toxin exposure (cadmium and mercury) was significantly reduced during the pandemic.

**Conclusions:**

The COVID-19 pandemic did not substantially alter growth, nutrition or immune function in infants and toddlers, though micronutrient intakes and toxin exposure shifted. Long-term monitoring is warranted.

Since the onset of the COVID-19 pandemic in late 2019, its far-reaching effects have significantly altered global economies, social structures and healthcare systems^(1,2)^. In response to the outbreak, many countries enforced lockdowns and restrictions on social activities, which dramatically impacted the lifestyles of pregnant women^(3,4)^. Foetuses and newborns, being particularly vulnerable, faced not only the direct risks associated with COVID-19 infection but also the indirect consequences stemming from disrupted healthcare services and increased social isolation. These disruptions have had lasting repercussions on infant health, potentially leading to delays in growth and development, malnutrition, immune dysfunction and mental health challenges.

Furthermore, lockdowns and supply chain disruptions during the pandemic could have significantly altered household food accessibility and dietary patterns. Such shifts are particularly critical for infants and toddlers, whose nutrient requirements are high for growth and development and whose diets are reliant on adult provision. Investigating nutritional intake during this period is therefore crucial to understanding the pandemic’s full impact on child health.

COVID-19 infection exerts its effects primarily through dysregulated immune responses, rather than through direct viral damage to organs^(5)^. The virus interferes with the innate immune system and alters the host’s immune response, with severe cases often progressing to multisystem inflammatory syndrome in neonates^(6)^. Additionally, viral infections like COVID-19 have been linked to various autoimmune diseases, including systemic rheumatoid arthritis, lupus erythematosus and multiple sclerosis^(7,8)^. However, it remains unclear whether exposure to COVID-19 during critical periods of foetal or neonatal development may have long-term consequences on immune system function^(9)^.

Neutrophils, monocytes, lymphocytes and platelets play crucial roles in immune responses by releasing inflammatory cytokines and autoantibodies. Inflammatory markers such as the neutrophil-to-lymphocyte ratio (NLR), lymphocyte-to-monocyte ratio (LMR), platelet-to-lymphocyte ratio (PLR) and systemic immune-inflammation index (SII) have gained recognition as reliable indicators of inflammation and immune status^(10–13)^.

This study aims to explore two primary questions: (1) How has the nutritional intake and physical growth of children born during the COVID-19 pandemic been affected? and (2) Do their immune systems function normally, or do they show signs of dysfunction, as indicated by inflammatory markers?

## Methods

### Study population

This analysis draws on data from the National Health and Nutrition Examination Survey (NHANES), an ongoing, nationally representative surveillance programme initiated in the early 1960s to monitor the health and nutritional status of the U.S. population. Its complex survey design ensures generalisability to the broader U.S. public. The publicly released data, organised in sequential cross-sectional cycles, encompass demographic information, dietary recalls, physical examination findings, laboratory tests and responses to health questionnaires. For comprehensive methodological details, please refer to the official NHANES website^(14)^.

Given that the first confirmed case of COVID-19 in the U.S. was reported in January 2020^(15)^, we defined the period from 2020 to 2021 as the COVID-19 pandemic period. This analysis included data from two NHANES cycles: 2017–2018 (pre-pandemic) and 2021–2023 (pandemic period). Specifically, we selected children aged 12–24 months from the 2021–2023 NHANES cycle who were born during the pandemic (i.e. between 2020 and 2021) and raised during this period. As the 2019–2020 NHANES cycle was disrupted by the pandemic, it was excluded from the analysis. The reference group consisted of children aged 12–24 months from the 2017–2018 cycle, representing those born prior to the pandemic. Although NHANES did not systematically collect COVID-19 infection status for all participants during the 2021–2023 cycle, the dataset remains representative of the U.S. paediatric population during the pandemic period, capturing a broad range of health and nutritional outcomes.

### Study variables

#### Covariates

Demographic variables included age, race, sex, maternal education level and the family income-to-poverty ratio (used as a proxy for socioeconomic status, categorised as < 1·85, 1·85–3·5 or > 3·5)^(16)^. Other covariates included parental marital status, household size, birth weight (categorised as < 2·5 kg and ≥ 2·5 kg) and breastfeeding history, as reported in NHANES reproductive history questionnaires. Breastfeeding data were derived from the question: ‘Was {Child Name} ever breastfed or fed breast milk?’. This question specifically pertained to the study child, ensuring the accuracy of the breastfeeding history variable used in our analysis.

#### Dietary intake assessment

Dietary intake data were collected by trained interviewers using the Automated Multiple-Pass Method, a standardised five-step 24-hour dietary recall protocol designed to enhance completeness and accuracy. Interviewers used standard measuring cups, spoons, rulers and a set of two- and three-dimensional food models to assist participants in estimating portion sizes, in accordance with NHANES protocol. Nutrient intakes were calculated using the USDA Food and Nutrient Database for Dietary Studies linked to the survey cycle^(17)^. A minimum reportable intake amount was not specified; all consumed foods and beverages were recorded. Nutritional intake variables included total energy, macronutrients (protein, carbohydrate, total fat, SFA, MUFA and PUFA and cholesterol), dietary fibre and key micronutrients (including vitamins A, C, D, E, K, B_1_, B_2_, B_6_, B_12_, total folate, calcium, phosphorus, magnesium, iron, zinc, copper, sodium, potassium, selenium and carotenoids).

#### Laboratory assessment

Laboratory measurements encompassed complete blood count, C-reactive protein, ferritin, transferrin receptor, RBC folate and blood levels of cadmium, mercury, selenium, manganese and 25-hydroxyvitamin D2 + D3. Blood cell counts (lymphocytes, neutrophils, platelets, monocytes, basophils, eosinophils), red cell distribution width, mean platelet volume (MPV) and Hb levels were measured using automated haematology analysers following standardised NHANES protocols, with blood cell counts expressed as ×10^3^/µL and Hb levels in g/dl^(18)^. Laboratory data with missing values were excluded from the statistical analysis.

#### Outcome definitions

The primary outcomes included birth weight, weight, length and BMI, calculated using the formula: BMI = weight (kg)/(height (m))^2^. Weight and length/height were directly measured by trained health technicians following standardised NHANES protocols, rather than being self-reported, ensuring high data quality. To enable valid comparisons across ages and cohorts, weight, length and BMI were converted to Z-scores based on the WHO Child Growth Standards. Z-scores for weight, length and BMI were calculated based on the WHO Child Growth Standards for children born during the pandemic^(19)^.

Inflammatory markers were calculated as follows: the SII was derived by multiplying platelet count by neutrophil count and dividing by lymphocyte count. The NLR was calculated as the neutrophil count divided by the lymphocyte count, while the PLR was calculated as the platelet count divided by the lymphocyte count. The LMR was derived by dividing the lymphocyte count by the monocyte count.

### Statistical analysis

All statistical analyses were conducted in accordance with the guidelines established by the Centers for Disease Control and Prevention (CDC), which account for NHANES’ complex, multistage cluster survey design. Each participant in NHANES is assigned a sample weight that reflects the number of individuals in the U.S. population represented by that participant. This weight adjusts for the unequal probability of selection, nonresponse and population control^(20)^.

To address missing data, we employed multiple imputation using a multivariate logistic regression model, iterating the process for ten cycles to generate a final imputed dataset. Statistical comparisons were performed employing *t* tests for continuous variables and chi-squared tests for categorical variables. Non-normally distributed continuous data are reported as median (IQR; p25–p75), while categorical data are expressed as proportions. The Kruskal–Wallis test was applied to evaluate differences across multiple groups.

Additionally, we compared physiological indicators (including weight, length, BMI, NLR, PLR, LMR and SII) between pre-pandemic and pandemic-born cohorts, stratified by sex. Data were transformed into a long format, and mean values with 95 % CI were calculated to illustrate differences. Local polynomial regression (LOESS) curves were used to examine trends over time. *t* tests were conducted to assess differences between birth cohorts and sexes, with *P*-values reported for each comparison.

All statistical analyses were conducted using R software (https://www.R-project.org, The R Foundation), with a two-sided *P*-value of less than 0·05 defining statistical significance.

## Results

### Demographic and socioeconomic characteristics

Table 1 summarises the demographic and socioeconomic characteristics of a weighted cohort of 7 401 768 children, divided into two birth cohorts: pre-pandemic (3 904 863 children) and pandemic (3 496 904 children). The cohort was weighted to ensure accurate representation of the sample distribution. The median age of the children was 18 months, with no significant difference between the pre-pandemic and pandemic groups (*P* = 0·060). Gender distribution was also similar between the cohorts, with 52 % boys and 48 % girls overall (*P* = 0·208).

**Table 1.** Demographic, socioeconomic and birth characteristics of the study population, stratified by birth cohort (pre-pandemic *v*. pandemic) Table 1 long description.

Characteristic	*n* ^*^			Birth cohorts				*P*-value^‡^
		Overall		Pre-pandemic		Pandemic		
		*n* 7 401 768^†^		*n* 3 904 863^†^		*n* 3 496 904^†^		
		*n*	%	*n*	%	*n*	%	
Age (months)								
Median	428	18·0		18·0		17·0		0·060
IQR		15·0–21·0		15·0–21·0		14·0–21·0		
Gender	428							0·208
Boys		3 829 197	52 %	2 141 223	55 %	1 687 973	48 %	
Girls		3 572 571	48 %	1 763 640	45 %	1 808 931	52 %	
Race/ethnicity	428							0·224
Non-Hispanic White		3 640 247	49 %	1 955 241	50 %	1 685 006	48 %	
Non-Hispanic Black		1 164 516	16 %	518 395	13 %	646 122	18 %	
Multi-Racial		744 500	10 %	452 160	12 %	292 340	8·4 %	
Mexican American		932 538	13 %	609 612	16 %	322 926	9·2 %	
Other Hispanic		919 966	12 %	369 456	9·5 %	550 510	16 %	
Household size								
Median	428	4·0		4·0		4·0		0·327
IQR		3·0–5·0		3·0–5·0		3·0–5·0		
Parental education level	428							0·286
Below high school		907 081	12 %	588 211	15 %	318 870	9·1 %	
High school		4 237 944	57 %	2 329 069	60 %	1 908 875	55 %	
College degree or higher		2 256 743	30 %	987 583	25 %	1 269 160	36 %	
Parents’ marital status	428							0·570
Married/Partnered		6 275 464	85 %	3 232 623	83 %	3 042 841	87 %	
Lost partner		504 426	6·8 %	301 996	7·7 %	202 430	5·8 %	
Single		621 878	8·4 %	370 245	9·5 %	251 633	7·2 %	
Poverty income ratio	428							0·020
< 1·85		3 216 246	43 %	2 021 210	52 %	1 195 035	34 %	
1·85∼3·50		2 040 013	28 %	907 077	23 %	1 132 936	32 %	
> 3·50		2 145 509	29 %	976 577	25 %	1 168 932	33 %	
Breastfeeding	428							0·269
Ever		6 242 591	84·2 %	3 388 675	86·4 %	2 853 916	82 %	
Never		1 159 177	16 %	516 189	13 %	642 988	18 %	
Birth weight (kilogram)	428							0·861
< 2·5		631 875	8·5 %	334 451	8·6 %	297 425	8·5 %	
2·5–4·0		6 251 262	84 %	3 273 020	84 %	2 978 243	85 %	
≥ 4·0		518 630	7·0 %	297 393	7·6 %	221 237	6·3 %	

* *n* not missing (unweighted).

† Median (IQR) for continuous; *n* (%) for categorical.

‡ Design-based Kruskal–Wallis test; Pearson’s X^2: Rao & Scott adjustment.

Regarding race/ethnicity, the cohort included 49 % Non-Hispanic White, 16 % Non-Hispanic Black, 10 % Multi-Racial, 13 % Mexican American and 12 % Other Hispanic children, with no significant differences observed between the two cohorts (*P* = 0·224). Household size remained consistent, with a median of four members in both groups. Parental education levels (*P* = 0·286) and marital status (*P* = 0·570) showed no significant differences. However, the poverty income ratio differed significantly between the cohorts (*P* = 0·020), with a higher proportion of children in the pre-pandemic cohort living below the poverty line (52 %) compared to the pandemic cohort (34 %). Breastfeeding rates were high across both cohorts, with 84 % of children being breastfed, and birth weight distributions showed no significant differences (*P* = 0·269 and *P* = 0·861, respectively). These findings suggest that while most demographic and socioeconomic characteristics remained stable, there was a notable reduction in income disparities during the pandemic.

### Dietary nutrient intake

There were no significant differences in energy, protein, carbohydrate, total sugar or fat intake between the two cohorts. The median energy intake was approximately 1035 kcal/d, with protein and carbohydrate intakes averaging 36·3 g/d and 125·2 g/d, respectively. However, specific nutrient intakes revealed significant differences. Vitamin A intake was significantly lower in the pandemic cohort (437·0 mcg/d) compared to the pre-pandemic cohort (540·0 mcg/d, *P* = 0·006). Conversely, vitamin C intake was higher during the pandemic (87·0 mg/d *v*. 62·8 mg/d, *P* < 0·001). Vitamin B_12_ intake also decreased significantly in the pandemic cohort (2·7 mcg/d *v*. 3·4 mcg/d, *P* = 0·009), and zinc intake was slightly lower (5·9 mg/d *v*. 6·3 mg/d, *P* = 0·045). In contrast, dietary iron intake did not differ between cohorts (*P* = 0·172), which is consistent with the absence of differences in iron status biomarkers (ferritin, transferrin receptor and Hb). No statistically significant differences were observed for any other nutrients (Table 2).

**Table 2.** Dietary nutrient intakes of children, stratified by birth cohort (pre-pandemic *v*. pandemic)

Characteristic	*n* ^*^			Birth cohorts				*P*-value^‡^
		Overall		Pre-pandemic		Pandemic		
		*n* 7 410 297^†^		*n* 3 753 772^†^		*n* 3 656 525^†^		
		Median	IQR	Median	IQR	Median	IQR	
Energy (kcal/d)	428	1035·5	786·5–1307·5	1006·0	765·5–1266·5	1057·0	810·0–1353·5	0·295
Protein (g/d)	428	36·3	20·2–48·0	36·5	19·2–48·3	36·3	21·0–47·3	0·754
Carbohydrate (g/d)	428	125·2	94·8–164·1	120·2	92·8–158·5	126·5	95·4–168·9	0·299
Total sugars (g/d)	428	71·1	56·9–92·7	72·4	57·1–92·7	69·9	56·9–92·1	0·970
Dietary fibre (g/d)	428	6·7	3·6–9·9	6·5	3·2–10·3	7·0	4·2–9·8	0·631
Dietary fibre (g/d)	428	42·2	32·0–52·3	42·3	30·6–51·6	40·5	32·1–52·5	0·736
Total saturated fatty (g/d)	428	16·9	12·4–20·5	16·7	11·8–20·8	16·9	13·3–19·8	0·923
Total monounsaturated fats (g/d)	428	12·9	9·7–17·5	13·0	9·7–17·3	12·9	9·7–18·0	0·932
Total polyunsaturated fatty (g/d)	428	7·8	5·8–11·0	7·8	5·6–10·9	7·7	5·9–11·4	0·703
Cholesterol (mg/d)	428	107·0	22·0–187·0	105·5	19·0–182·0	110·0	29·5–191·0	0·731
Vitamin E (mg/d)	428	5·6	3·9–7·9	5·5	3·8–8·3	5·8	4·2–7·4	0·968
Vitamin A (mcg/d)	428	503·0	347·0–702·0	540·0	404·0–743·5	437·0	332·5–624·5	0·006
Alpha-carotene (mcg/d)	428	30·0	3·0–273·5	30·0	2·5–277·5	36·5	3·0–249·5	0·834
Beta-carotene (mcg/d)	428	346·5	99·0–1352·0	333·5	83·5–1552·0	346·5	103·5–1345·5	0·963
Vitamin B_1_ (mg/d)	428	0·9	0·6–1·2	0·9	0·6–1·2	0·9	0·6–1·2	0·583
Vitamin B_2_ (mg/d)	428	1·3	1·0–1·6	1·3	1·0–1·7	1·3	1·0–1·6	0·598
Vitamin B_6_ (mg/d)	428	0·9	0·6–1·3	0·9	0·6–1·3	0·9	0·7–1·3	0·943
Total folate (mcg/d)	428	150·0	116·0–228·5	156·0	111·5–239·0	140·5	117·5–220·5	0·696
Vitamin B_12_ (mcg/d)	428	3·0	2·0–4·2	3·4	2·0–4·6	2·7	1·9–3·8	0·009
Vitamin C (mg/d)	428	71·6	45·6–100·7	62·8	39·0–89·1	87·0	57·3–108·9	< 0·001
Vitamin D (D2 + D3) (mcg/d)	428	7·2	5·5–9·8	7·3	6·1–9·8	7·0	5·2–9·0	0·292
Vitamin K (mcg/d)	428	43·8	29·2–64·0	42·0	31·2–60·8	50·4	27·3–65·7	0·391
Calcium (mg/d)	428	846·5	603·0–1058·0	875·5	617·0–1101·0	820·5	602·0–1017·5	0·258
Phosphorus (mg/d)	428	791·5	462·0–1046·0	795·5	436·5–1046·0	786·0	474·0–1040·5	0·938
Magnesium (mg/d)	428	147·0	92·5–193·0	146·0	86·5–192·5	147·0	104·0–195·5	0·383
Iron (mg/d)	428	9·4	6·0–13·3	9·8	6·5–14·3	8·8	5·3–12·9	0·172
Zinc (mg/d)	428	6·1	4·8–7·7	6·3	4·9–8·5	5·9	4·8–7·2	0·045
Copper (mg/d)	428	0·6	0·4–0·7	0·6	0·4–0·7	0·6	0·4–0·7	0·984
Sodium (mg/d)	428	1226·0	420·0–1598·0	1149·5	331·5–1643·5	1242·5	463·0–1578·0	0·378
Potassium (mg/d)	428	1475·5	1037·0–1958·0	1411·0	1011·5–1984·0	1521·0	1044·5–1836·5	0·796
Selenium (mcg/d)	428	41·6	23·8–57·5	41·7	22·1–60·4	41·6	25·0–53·0	0·762
Moisture (g/d)	428	1115·2	900·7–1366·5	1106·4	866·1–1317·5	1115·2	900·7–1387·3	0·379

* *n* not missing (unweighted).

† Median (IQR) for continuous; *n* (%) for categorical.

‡ Design-based Kruskal–Wallis test.

### Laboratory results

Most laboratory parameters, including leukocyte counts, lymphocytes, neutrophils, basophils, eosinophils and monocytes, showed no significant differences between the two cohorts. Hb levels were slightly lower in the pandemic cohort, but this difference was not statistically significant (*P* = 0·086). Platelet count and red cell distribution width also did not differ significantly between the cohorts.

Notably, environmental toxin exposure was significantly reduced in the pandemic cohort. Blood cadmium levels were lower in the pandemic cohort (0·05 µg/l) compared to the pre-pandemic cohort (0·07 µg/l, *P* < 0·001), and blood mercury levels were similarly reduced (0·12 µg/l *v*. 0·20 µg/l, *P* = 0·002). Other biomarkers, such as blood selenium, manganese and vitamin D, did not show significant differences between the cohorts (Table 3).

**Table 3.** Laboratory indicators of nutritional and health status, stratified by birth cohort (pre-pandemic *v*. pandemic)

Characteristic	*n* ^*^			Birth cohorts				*P*-value^‡^
		Overall		Pre-pandemic		Pandemic		
		*n* 7 386 569^†^		*n* 3 727 003^†^		*n* 3 659 567^†^		
		Median	IQR	Median	IQR	Median	IQR	
Complete blood count								
Leukocytes (10^‡^/uL)	174	8·60	7·70–10·00	8·80	7·70–10·60	8·50	7·90–9·60	0·530
Lymphocytes (10^‡^/uL)	174	5·00	4·10–5·90	4·90	4·10–6·20	5·00	4·20–5·60	0·911
Neutrophils (10^‡^/uL)	174	2·60	1·90–3·60	2·60	1·80–3·90	2·50	2·10–3·40	0·748
Basophils (10^‡^/uL)	174	0·10	0·00–0·10	0·10	0·00–0·10	0·00	0·00–0·10	0·768
Eosinophils (10^‡^/uL)	174	0·20	0·10–0·30	0·20	0·10–0·30	0·20	0·20–0·30	0·517
Monocytes (10^‡^/uL)	174	0·70	0·60–0·90	0·70	0·60–0·90	0·60	0·60–0·70	0·166
Hb (g/dl)	174	12·40	11·80–12·80	12·40	12·00–12·90	12·10	11·60–12·60	0·086
Platelets (10^‡^/uL)	174	354	295–408	342	292–407	356	295–408	0·689
Red cell distribution width (%)	174	13·80	13·20–14·50	13·80	13·20–14·50	14·00	13·00–14·60	0·737
Mean platelet volume (fL)	174	7·00	6·60–7·50	7·00	6·60–7·50	6·80	6·50–7·50	0·251
C-reactive protein (mg/l)	134	0·39	0·26–0·82	0·40	0·28–0·82	0·34	0·15–0·77	0·475
Ferritin (ng/ml)	152	30	20–43	28	19–44	30	20–37	0·827
Transferrin receptor (mg/l)	144	4·00	3·48–4·45	3·94	3·49–4·35	4·08	3·44–4·56	0·482
RBC folate (ng/ml)	102	481	411–578	481	417–561	481	404–600	0·928
Blood cadmium (ug/l)	171	0·07	0·05–0·07	0·07	0·07–0·07	0·05	0·05–0·07	< 0·001
Blood mercury (ug/l)	171	0·20	0·19–0·31	0·20	0·20–0·33	0·12	0·12–0·26	0·002
Blood selenium (ug/l)	171	156	139–171	158	143–172	154	138–164	0·245
Blood manganese (ug/l)	171	11·2	9·1–13·4	11·2	8·8–13·4	11·2	9·9–13·2	0·774
25-hydroxyvitamin D2 + D3 (nmol/l)	146	78	64–89	78	67–93	77	63–86	0·523

* *n* not missing (unweighted).

† Median (IQR) for continuous; *n* (%) for categorical.

‡ Design-based Kruskal–Wallis test.

### Growth metrics

**Figure 1.**
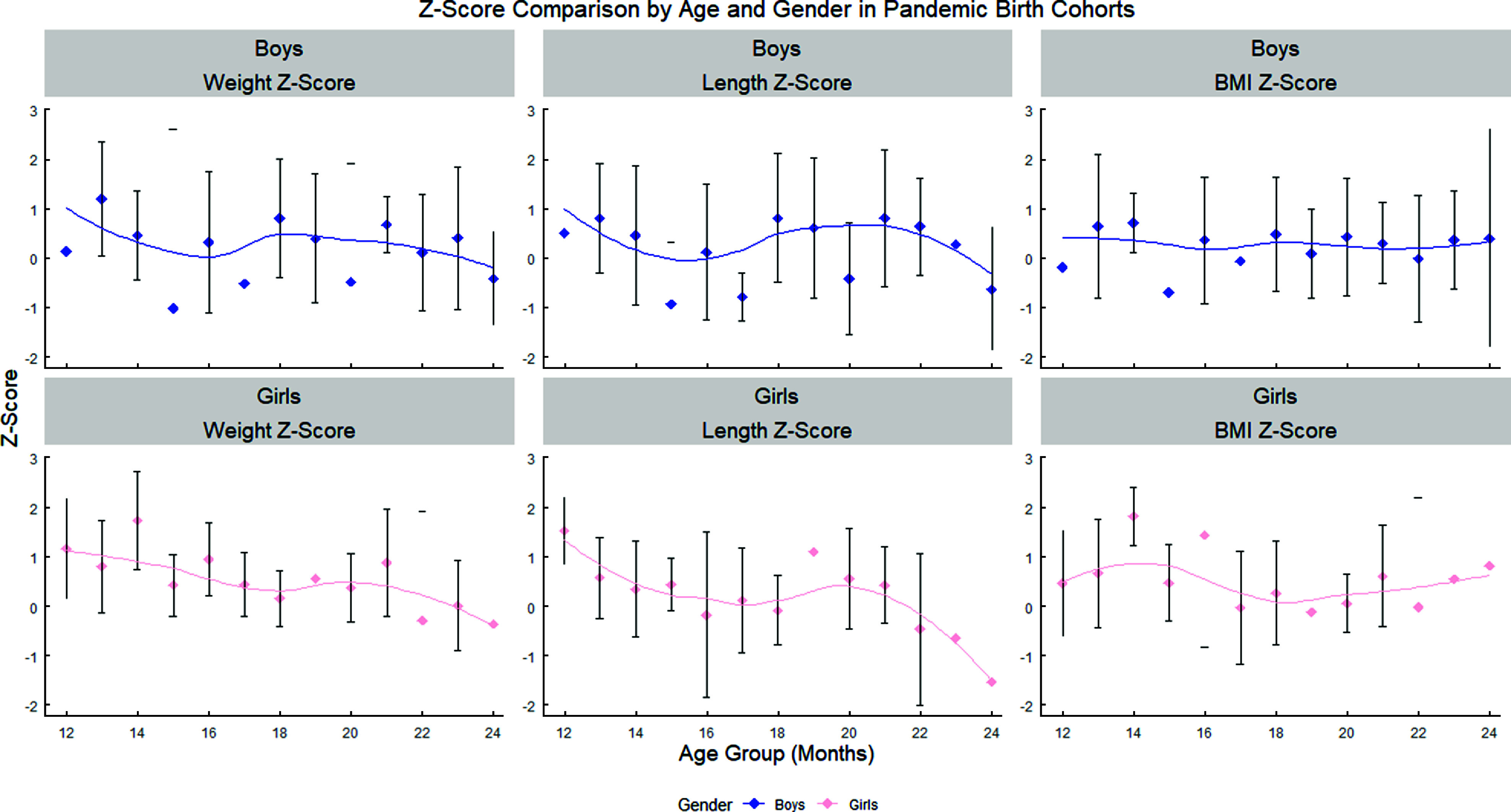
Figure 1 long description. Z-Score comparison of weight, length and BMI by age and gender in pandemic birth cohorts.

**Figure 2.**
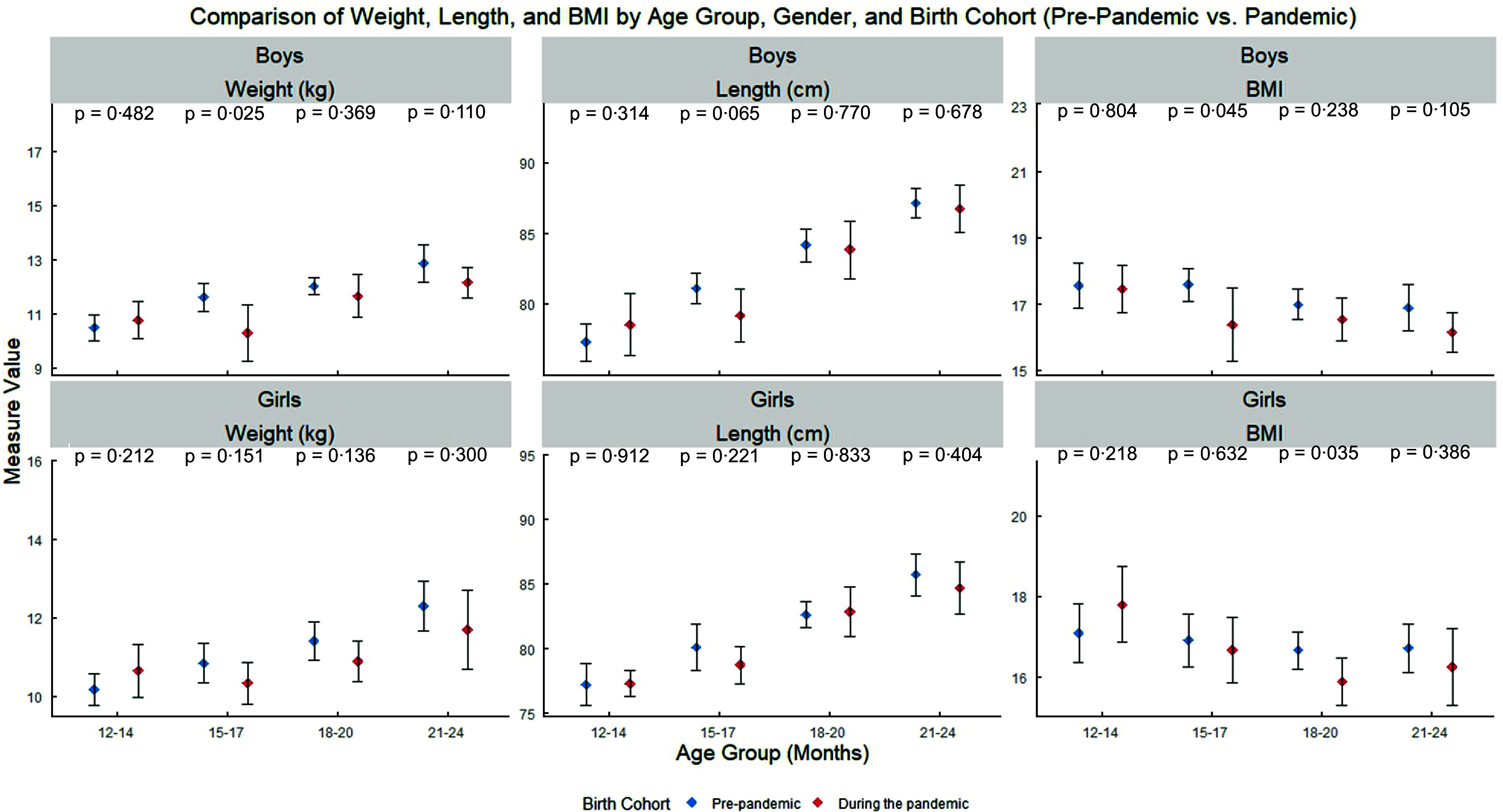
Comparison of weight, length and BMI by age group, gender and birth cohort (pre-pandemic *v.* pandemic).

Figures 1 and 2 illustrate Z-scores for growth metrics, including weight-for-age, length-for-age and BMI-for-age, for boys and girls across age groups in the pre-pandemic and pandemic birth cohorts. The overall mean (sd) Z-scores for the pandemic cohort were as follows: weight-for-age: (0·52 (sd 1·00)), length-for-age: (0·37 (sd 1·10)) and BMI-for-age: (0·44 (sd 1·00)). All mean Z-scores were within the normal range (–2 to +2), suggesting that growth trajectories in both cohorts adhered to expected developmental patterns, with only minor fluctuations.

Figure 2 provides a direct comparison of growth metrics between the two cohorts. Boys born during the pandemic exhibited significantly lower BMI at 15–17 months (*P* = 0·045), while no significant differences were observed in weight or length across other age groups. For girls, BMI was significantly lower at 18–20 months (*P* = 0·035), with no notable differences in weight or length. Overall, most *P*-values indicated minimal differences between the cohorts, suggesting that the pandemic had a limited impact on growth metrics.

### Inflammatory markers

**Figure 3.**
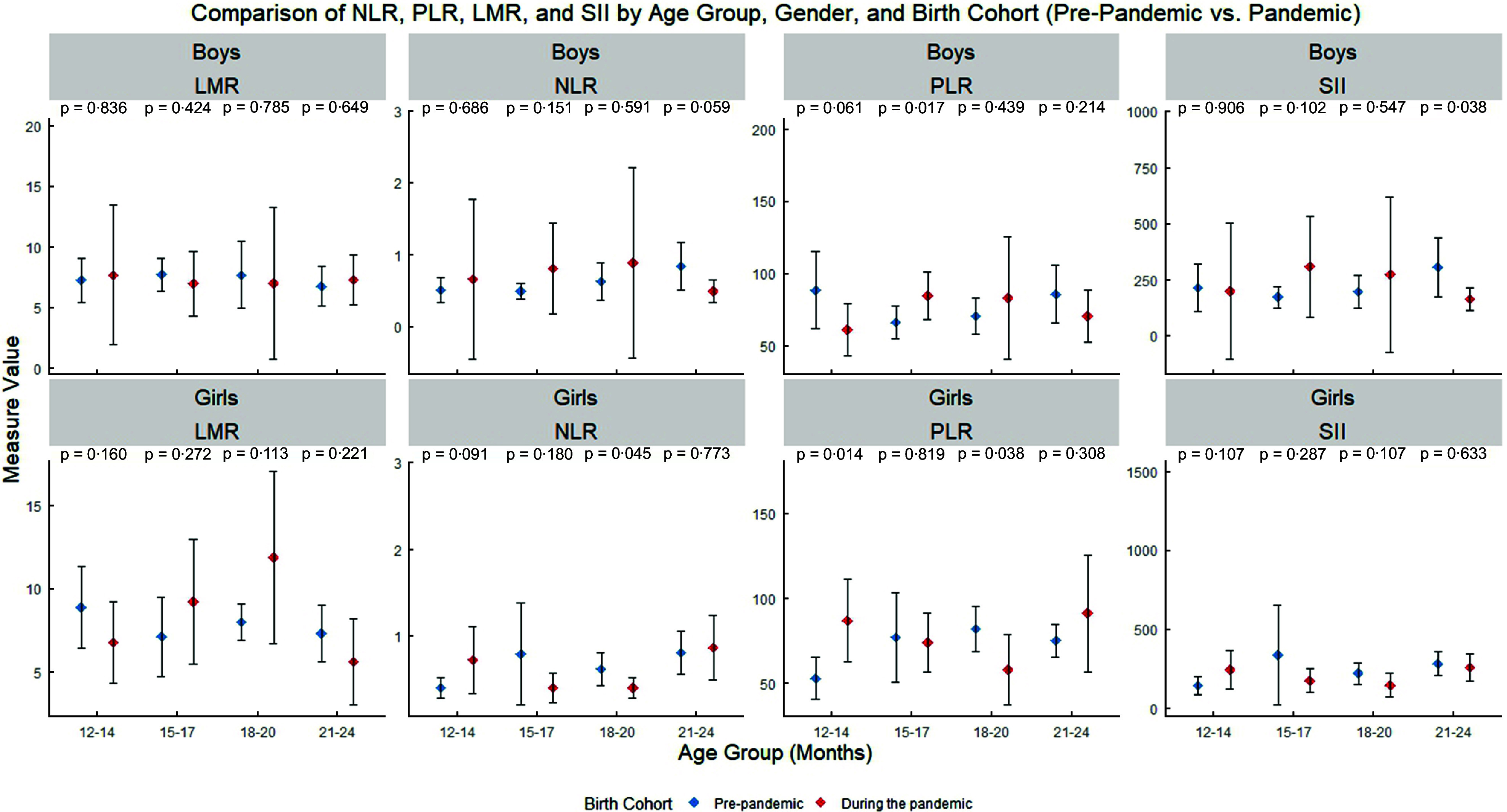
Figure 3 long description. Comparison of NLR, PLR, LMR and SII by age group, gender and birth cohort (pre-pandemic *v.* pandemic).

Figure 3 compares inflammatory markers, including the LMR, NLR, PLR and SII, across age groups, gender and birth cohorts.

For boys, no significant differences were observed between the two cohorts for most inflammatory markers, except for PLR at 15–17 months (*P* = 0·017) and SII at 21–24 months (*P* = 0·038). In girls, significant differences were observed for PLR at 12–14 months (*P* = 0·014) and 18–20 months (*P* = 0·038). NLR at 18–20 months also approached significance (*P* = 0·045). These findings suggest some variations in immune profiles between the two cohorts. However, most values remained within the normal range, indicating that the overall immune status of children in both cohorts was largely unaffected by the pandemic.

## Discussion

The present analysis utilised data from the NHANES, a large, diverse dataset that is representative of the U.S. population^(16,21)^. By including newborns from community settings, the findings can be generalised to the broader U.S. population^(22)^. Our study demonstrated that, overall, most demographic, nutritional, laboratory and growth indicators for children remained stable before and during the COVID-19 pandemic. While certain micronutrient intakes and a few inflammatory markers exhibited notable differences, the overall development and health status of children were largely within normal and expected ranges.

COVID-19 infection has the potential to directly impact neonatal growth and development, primarily through systemic inflammation. Severe COVID-19 cases are associated with cytokine storms, which lead to elevated levels of pro-inflammatory cytokines, such as IL-6 and tumour necrosis factor-alpha^(23,24)^. These cytokines can suppress the function of insulin-like growth factor-1, a critical regulator of bone and muscle growth^(25)^. Disruption of insulin-like growth factor-1 activity may result in growth delays, particularly in cases of severe or prolonged inflammation^(26)^.

Additionally, symptoms associated with COVID-19 – such as fever, respiratory distress and feeding difficulties – can adversely affect nutritional intake, further exacerbating metabolic stress. Neonates, in a critical phase of rapid growth, require adequate energy and nutrient intake to support weight gain and linear growth. Insufficient energy intake during this period can lead to negative energy balance, ultimately suppressing growth. Deficiencies in essential micronutrients, such as zinc and iron, during key growth periods are linked to impaired cognitive and motor development^(27–31)^. Bone development may also be compromised, as infections can disrupt calcium and phosphorus metabolism, both of which are essential for bone mineralisation^(32,33)^. Chronic infections may further impair vitamin D status, delaying skeletal growth and potentially contributing to long-term developmental challenges^(34)^.

Beyond the direct effects of SARS-CoV-2 infection, the broader context of the pandemic – including lockdowns, work-from-home policies and reduced outdoor activities – may have exerted indirect influences on child health. On one hand, restricted access to healthcare services, disruptions in food supply chains and diminished availability of nutritional support programmes may have negatively impacted maternal and neonatal health outcomes^(35,36)^. On the other hand, increased parental presence at home could have facilitated more attentive caregiving and closer monitoring of dietary intake, potentially mitigating adverse nutritional outcomes. Similarly, the significant reduction in exposure to environmental toxins such as cadmium and mercury – likely resulting from decreased industrial and traffic-related pollution during the pandemic – may have contributed to a less pro-inflammatory environment. Vulnerable populations, particularly those in resource-limited settings, may have been disproportionately impacted by these disruptions^(37,38)^.

Despite these challenges, our study found no significant differences in most nutrient intakes between children born before and during the pandemic. Macronutrient intake, including energy, protein and fat, as well as trace elements and vitamins, remained stable. Similarly, laboratory indicators of nutritional and health status, such as Hb, ferritin and blood levels of 25-hydroxyvitamin D2 + D3, showed no significant differences. The observed shift towards higher vitamin C intake coupled with lower intakes of vitamin A and B_12_ (Table 2) may reflect changes in dietary patterns and food accessibility during the pandemic. The elevated vitamin C intake likely indicates increased parental awareness of, or preference for, foods perceived to support immune function. Conversely, the decrease in vitamins A and B_12_ could be attributable to disruptions in the supply chain for certain fortified foods or animal products or to broader shifts in household dietary habits. While these differences are noteworthy, they did not significantly alter overall growth trends, as both groups maintained normal Z-scores for weight, length and BMI.

Although physical growth was not significantly affected, increasing evidence suggests that COVID-19 may induce autoimmune phenomena through immune system dysregulation^(39)^. Overactivation of the immune system can lead to autoimmune inflammation, which disrupts normal immune responses and results in immune system imbalance^(5,40)^. This dysregulation not only impairs the body’s ability to defend against infections but may also increase the risk of developing various chronic conditions, such as autoimmune diseases^(6)^, cancer^(41,42)^, diabetes^(43)^, CVD^(44,45)^ and metabolic syndrome^(46,47)^. To assess changes in immune function, inflammatory markers such as the NLR, LMR, PLR and the SII are widely recognised as reliable indicators of inflammation and immune status^(10)^.

In interpreting the inflammatory markers in our study, it is essential to consider the likelihood of widespread SARS-CoV-2 exposure within the pandemic cohort. Although individual-level infection data were not available in NHANES, the high community transmission during the study period – especially with the dominance of the highly transmissible Omicron variant – suggests that most U.S. children, including those in our sample, had been infected. National seroprevalence studies support this, estimating that over 90 % of children had acquired SARS-CoV-2 antibodies by the end of 2022^(48)^. Against this backdrop of substantial viral exposure, the only minor and clinically non-significant fluctuations observed in NLR, PLR, LMR and SII in our study are particularly reassuring. These findings suggest that, at a population level, the innate and adaptive immune systems in infants and toddlers exhibited considerable resilience, effectively re-establishing homeostasis after widespread viral exposure without evidence of persistent inflammatory dysregulation.

Moreover, a substantial body of evidence indicates that inflammatory markers are closely associated with growth and development in children. Studies have linked these markers to malnutrition^(49)^, overweight^(50,51)^, bronchopulmonary dysplasia^(52)^ and retinopathy of prematurity^(53)^ – all common issues in neonates. In our study, inflammatory markers showed minor variations in certain age groups of pandemic-born children but remained within normal ranges overall. These findings suggest that the immune function of pandemic-born children was minimally impacted, and the likelihood of future growth abnormalities remains low.

While these findings are encouraging, the study has some limitations. First, its cross-sectional design does not allow for the establishment of causal relationships. To simulate a longitudinal approach, we compared growth indicators across children of different ages, but the lack of repeated measurements on the same individuals prevents the evaluation of individual changes over time. Second, the age range of the study participants was relatively narrow, and the absence of long-term follow-up data limits the ability to assess future growth and developmental trajectories. Third, laboratory data used in this study were derived from a single blood test. Serial measurements could provide a more comprehensive understanding of changes in nutritional and inflammatory markers over time. Fourth, data on individual COVID-19 infection status (including maternal infection during pregnancy) were not collected in NHANES, precluding a sub-analysis comparing directly infected and non-infected children. However, given the high seroprevalence in the paediatric population during the study period, the pandemic cohort overall represents a group with substantial viral exposure. Future prospective studies with serial serological testing would be valuable to correlate the timing and severity of infection with long-term growth and immune outcomes.

## Conclusion

In conclusion, the COVID-19 pandemic did not appear to have a significant negative impact on the physical growth, nutritional intake, or immune function of children born during this period. Although minor variations were observed in certain inflammatory markers and nutrient intakes, the overall health status of these children remained within normal ranges. Future studies, particularly prospective research, are needed to evaluate the long-term effects of the pandemic on child development and health outcomes.

## Table 1. Long description

The table presents demographic and socioeconomic characteristics of a weighted cohort of 7401768 children, divided into pre-pandemic (3904863 children) and pandemic (3496904 children) birth cohorts. The table has 428 rows and 10 columns. Column headers are Characteristic, n, Overall n, Overall percent, Pre-pandemic n, Pre-pandemic percent, Pandemic n, Pandemic percent, and P-value. Row labels include Age (months), Gender, Race/ethnicity, Household size, Parental education level, Parents’ marital status, Poverty income ratio, Breastfeeding, and Birth weight (kilogram). Each row provides specific data points for each characteristic across the overall, pre-pandemic, and pandemic cohorts, along with corresponding P-values indicating statistical significance. Notable trends include similar gender distribution, with 52% boys and 48% girls overall, and a median age of 18 months with no significant difference between cohorts. Race/ethnicity distribution shows Non-Hispanic White at 49%, Non-Hispanic Black at 16%, Multi-Racial at 10%, Mexican American at 13%, and Other Hispanic at 12%. Household size has a median of 4. Parental education level is predominantly high school (57%), followed by college degree or higher (30%), and below high school (12%). Parents’ marital status shows 85% married/partnered, 6.8% lost partner, and 8.4% single. Poverty income ratio indicates 43% below 1.85, 28% between 1.85-3.50, and 29% above 3.50. Breastfeeding is reported in 84.2% of cases. Birth weight is predominantly between 2.5-4.0 kilograms (84%).

Navigate back to Table 1..

## Figure 1. Long description

The image contains six line graphs comparing Z-scores of weight, length, and BMI by age and gender in pandemic birth cohorts. The graphs are divided into two rows, with the top row representing boys and the bottom row representing girls. Each row contains three graphs: weight Z-score, length Z-score, and BMI Z-score. The x-axis for all graphs represents age groups in months, ranging from 12 to 24 months. The y-axis represents the Z-score, ranging from -2 to 3. Each graph includes data points with error bars and a trend line. Panel A (top left) shows the weight Z-score for boys, Panel B (top middle) shows the length Z-score for boys, and Panel C (top right) shows the BMI Z-score for boys. Panel D (bottom left) shows the weight Z-score for girls, Panel E (bottom middle) shows the length Z-score for girls, and Panel F (bottom right) shows the BMI Z-score for girls. The trend lines indicate variations in Z-scores over time for each gender and measurement type.

Navigate back to Figure 1..

## Figure 3. Long description

The image contains eight scatter plots comparing NLR, PLR, LMR, and SII by age group, gender, and birth cohort during pre-pandemic and pandemic periods. Each plot is divided into two rows for boys and girls, with four columns representing different measures: LMR, NLR, PLR, and SII. The x-axis of each plot represents age groups in months (12-14, 15-17, 18-20, 21-24), and the y-axis represents the measure value. Each plot includes data points for pre-pandemic (blue) and during the pandemic (red) cohorts. Panel A (Boys LMR) shows LMR values with p-values indicating statistical significance. Panel B (Boys NLR) shows NLR values with corresponding p-values. Panel C (Boys PLR) shows PLR values with p-values. Panel D (Boys SII) shows SII values with p-values. Panel E (Girls LMR) shows LMR values with p-values. Panel F (Girls NLR) shows NLR values with p-values. Panel G (Girls PLR) shows PLR values with p-values. Panel H (Girls SII) shows SII values with p-values. Each plot includes error bars representing variability in the data.

Navigate back to Figure 3..
